# May We Strengthen the Human Natural Defenses with Bacterial Lysates?

**DOI:** 10.1097/1939-4551-3-S2-S17

**Published:** 2010-08-15

**Authors:** Elisa Villa, Valentina Garelli, Fulvio Braido, Giovanni Melioli, Giorgio Walter Canonica

**Affiliations:** 1 Allergy Respiratory Disease Department, Department of Internal Medicine (DiMI), University of Genova, Italy; 2 Central Laboratory of Analysis, Giannina Gaslini Institute, Genoa, Italy

**Keywords:** bacterial lysates, airways infection, immunology

## Abstract

During the last twenty years bacterial lysates have gained a new interest and their use has obtained a progressively larger consensus in the medical practice. They are commonly used as immunomodulators, in order to up-regulate immune responses against infectious damages. As a matter of fact, the role of these lysate seems relevant in upper and lower respiratory tract infections prevention, frequently observed both in paediatric and elder ages, and which represent a relevant problem also in terms of socio-economical implications. The effects of bacterial lysates as immunostimulatory agents have become the central point of many studies. The aim of those in vivo and in vitro studies was to understand and evaluate the capacity of this kind of treatments to create a better answer of the immune system against microbial infections, eventually leading to a reduction in their number. All the in vivo and in vitro findings analyzed support the evidence that bacterial lysates are powerful inducers of a specific immune response against bacterial infections. Both in paediatric and adult clinical trials, a positive trend has been found in terms of overall reduction of infection rates and duration, beneficial effect on symptoms, reduction in antibiotics use and possibility to improve the patient's quality of life in several diseases. Further well-designed trials in terms of blinding and randomization procedures and including a higher number of patients, selected according to the disease and its severity, are needed.

## INTRODUCTION

Bacterial lysates have recently gained a new interest and their use has obtained a progressively larger consensus in the medical practice. Both researchers and medical doctors are lately focusing on the use of bacterial lysates for several reasons. Recurrent infections, in particular to upper and lower respiratory tract, are frequently observed in pediatric and elder ages and represent a relevant problem, because they have important socio-economical implications. At the moment, a large spectrum of different therapeutic options is available, but it is necessary to consider that the infectious episodes may derive from several physio-pathologic conditions. The immune system in children, for instance, is often relatively ineffective, in particular concerning the antibody responses to infectious stimuli such virus or bacteria. On the other hand, in elder subjects a general process of immune senescence is commonly observed, consisting in a global reduction of the number and the activity of the immune cells.^1 ^To increase the immune system reactivity toward the pathogens, in particular the bacteria, different therapeutic strategies can be chosen, from antibiotic prophylaxis to therapies based on vaccinations through the use of microbial origin preparations. But what really are bacterial vaccines?

Bacterial lysates were introduced in the 1970s, when the concept of the bacteria-derived immunomodulators appeared; they are mixtures of bacterial antigens derived from different microbic species, according to the considered extract. Antigens are obtained after a mass culture of reference bacterial strains, using a chemical (PCBL) or mechanical (PMBL) cellular lysis and lyophilization, then mixed in the same proportions; excipients are added to prepare the tablets. More often the extracts include different species (polyvalent extracts), such as *Staphylococcus Aureus, Streptococcus Viridans, S. Pneumoniae, S. Pyogenes, Klebsiella Pneumoniae, K. Ozenae, Moraxella Catarrhalis, Haemophilus Influenzae*, in amounts of billions of these bacteria.,^2^

PMBL are produced through a process that preserves the structure of the bacterial antigens and consists in: lysis in vitro, fractionation of bacterial bodies and/or supernatant and finally attainment of the particulate antigens. Because of their immunogenic capabilities, increasing importance is given to the significant effects that these extracts present in both experimental and clinical contexts, such as the prevention and the treatment of recurrent upper and lower respiratory airways infections. Nevertheless, PCBL are obtained through chemical alkaline substances and processes that may determine proteins denaturation, with a consequent lower immunogenicity of the antigens themselves. 

Bacterial vaccines commonly represent a prophylactic and therapeutic strategy for sequelae to bacterial or viral infections (ie, cold and influenza), acute and chronic bronchitis,^3,4 ^anginas, pharyngitis, tonsillitis, laryngitis, rhinitis, sinusitis, and otitis^5,6^; they are also indicated in case of infections resistant to common antibiotic treatments. Recently, bacterial lysates have been supposed to be useful also in preventing chronic obstructive pulmonary disease (COPD) exacerbations, defined as a worsening in the patient's, with limited airflows, baseline dyspnoea, cough, and/or sputum beyond day-to-day variability.^7,8 ^Research efforts have been recently directed to this drug class for many reasons: common treatment strategies are frequently unsuccessful, even though modern medicine offers several possibilities; antibiotics are not necessary during the nonacute phase of the disease; anti-inflammatory drugs are rarely effective and corticosteroids only partially modify the natural history of the disease.^9^

## MOLECULAR AND IMMUNOLOGIC MECHANISMS OF ACTION

Polyvalent bacterial lysates are commonly used as immunomodulators, to up-regulate immune responses against infectious damages. Mucosal surfaces, including those of gastrointestinal, respiratory, and urogenital tracts, represent the first mechanical barrier against the invasion of bacterial, viral and parasitic pathogens.^10,11 ^The potentiation of both specific and nonspecific immune responses against bacteria can be reached with the administration of polyvalent bacteria lysates as immunostimulators, eliciting protecting effects. When a pathogen succeeds in overcoming the epithelial barrier, it is recognized by the resident tissue leukocytes that, together with the epithelial cells, recall phagocytes cells (macrophages and neutrophils). These cells may directly interact with bacterial membrane receptors recognizing the pathogen-associated molecular patterns (eg, endotoxin identify Gram negative bacteria, while peptidoglycans identify the Gram positive ones) and unequivocally correlated with the nature of the pathogen; these receptors are called PRR (Pattern Recognition Receptors). Otherwise, phagocytes may recognize the pathogen indirectly, through receptors interacting with proteins that opsonize the bacteria, such as antibodies or proteins of the complement cascade.^12 ^The activated phagocyte cells show a global increase in their cytotoxic activity, thus leading to the killing and the elimination of the pathogen itself. In this immunologic setting, also dendritic cells (DCs) play an important role: as a matter of fact, they represent a link between innate and specific immune responses. DCs have the task to capture bacterial agents, transport them to loco-regional lymph-nodes and present specific antigens to T lymphocytes. During the presentation phase, the DCs express costimulating molecules, such as CD83, CD80, and CD86, and produce different cytokines that stimulate the activation of T cells and determine their polarization in T_H_1-cells or T_H_2-cells.^13^

To improve the immune defenses toward microbial antigens, a strategy may be directed to DCs: potentiating their immune activity, in fact, it is also possible to promote the activation of both lymphocytes and phagocytes cells.

Parental vaccines do not seem to determine a mucosal protection against pathogens, while oral, intranasal, and sub-lingual immunization can stimulate an antigen-specific immunoglobulins secretion at the application site, that results in local immune protection. Oral applications of microbial antigens have been shown to induce dissemination of lymphoid cells from the gut-associated lymphoid tissue to distant mucosa-associated effector sites, such as respiratory and genitourinary tracts and different excretory glands, where these elements proliferate and differentiate in effector cells.

The very first step in the immune-activation resulting from PMBL is the recognition of the microbeparticles by a specific group of cellular receptors, called Toll-Like Receptors (TLRs), expressed on the surface of monocytic-macrophage membranes.^14 ^It is a matter of fact that TLRs can directly interact with microbial ligands and this recognition may occur either outside [through the activation of receptors by lipopolysaccharide (LPS), lipopeptides/lipoproteins (LP), lipoteichoic acid (LTA) and peptidoglycan (PG)] or inside the cell (through microbe-specific PAMPs, such as double-stranded (ds) or single-stranded (ss) RNA and bacterial DNA with CpG motifs. The interaction between bacterial structures and TLRs results in the activation of resting monocytes, thus differentiating in mature DCs, able of playing the role of professional antigen presenting cells. This mechanism is necessary to obtain an effective immune-stimulation through the use of PMBL: the bacterial antigens included are able to activate monocytic-macrophagic cells in the submucosa, inducing the differentiation of immature dendritic cells in mature ones; this step then results in a stimulation of the T cell compartment, with significant induction and improvement of the T Helper cells functioning.

PMBL may also induce B cells to differentiate in plasmacells able to generate antibodies specifically directed to the antigens themselves; particular attention is focused on IgA antibodies, that allow the pathogen opsonization and the after phagocytosis and killing mediated by professional immune competent cells. Secretory IgA (sIgA) have a strategic position on mucosal surfaces and represent one of the first protective factors of the specific immune system against invading pathogens. Several studies have demonstrated that the administration of bacterial lysates induces the production of mucosal antigen-specific IgA at the application site and in the secretions, resulting in a local protection against bacterial infections.

Ismigen, a PMBL preparation of 8 different bacterial strains tested in vitro has shown to induce the activation of DCs, leading to a significantly higher expression of costimulating membrane molecules (CD80, CD83, CD86).^15 ^Furthermore, another study^16 ^demonstrated that this PMBL had several immunologic effects consisting in:

1. Activation of the IL-2 receptor (*IL-2R-ॅ*) on different lymphocyte subsets (B, CD4^+ ^T, and CD8^+ ^T cells) involved both in humoral and cellular immune responses (IL-2, in an autocrine manner, is essential for the clonal expansion of proliferating cells);

2. Induction of cytokine synthesis (*IL-2*, *IL-10*, *IL-12*, *IFN-ă*) in immune competent cells, promoting regulating immune responses;

3. Generation of CD4^+ ^and CD8^+ ^effector T cells.

The same study group analyzed the effect, in vivo, of the PMBL (orally administrated) in preventing recurrent infections of the upper respiratory tract (URTIs) in a group of patients with a medical history of URTI recurrence.^17 ^Clinical results were correlated to the levels of IgM memory B cells and the expression of the activation marker CD25 in peripheral blood lymphocytes using the flow cytometric method, before and at 1 and 3 months after the treatment. PMBL have shown to exert a therapeutic and preventing effect in acute and recurrent infections of the upper airways, which is significantly correlated with the activation and enhancement of both IgM memory B lymphocytes (*CD24*^+^*/CD27^+ ^*cells) and IL-2 receptor-expressing lymphocytes (*CD25*^+ ^cells) involved either in humoral or cellular immunity.

These results provides the capability of the investigated PMBL to elicit both innate and specific immune responses, as demonstrated by the significant stimulation of the expression of cellular activation markers, at different stages. In general, these findings suggest that the immunoprotective role of bacterial lysates observed in human beings are related to their immunomodulatory effect on the responses of different immune competent cells, either by direct cellular activation or through the generation and activation of immune effector cells, and on the complex intercellular network of cytokines production and signaling pathways.

## TRIALS AND EFFICACY OF BACTERIAL LYSATES

During the last 20 years the efficacy of bacterial lysates as immunostimulatory agents has become the central point of many clinical trials. The aim of those studies was to understand and evaluate the capacity of this kind of treatments to create a powerful answer of the immune system against microbial infections, eventually leading to a reduction in their number.

These studies are quite heterogeneous. There are many differences between the individuals enrolled concerning their age (pediatric vs adult patients) and their suffering diseases, including recurrent upper and lower airways infections, acute and chronic bronchitis, rhinosinusitis, and COPD.^18^

We will show and discuss briefly the results of these RCTs, to evaluate the future perspectives of using bacterial lysates in the field of respiratory diseases but it is important to point out that the use of bacterial extracts has been recently considered also in the treatment of other diseases besides respiratory illnesses, such as rheumatoid arthritis,^19 ^type 1 diabetes mellitus,^20 ^allergic rhinitis, asthma,^21,22 ^chronic sinusitis,^23 ^acute recurrent diverticulitis,^24 ^chronic colitis,^25,26 ^and inflammatory bowel disease,^27 ^chronic and recurrent urinary tract infections,^28 ^periodontitis.^29 ^Furthermore, different studies investigate the effect of bacterial lysates on human lymphocytes and macrophages functions in vitro and in vivo, for a better understanding of the mechanisms that support their clinical efficacy and an improvement of their use in both infectious and noninfectious pathologies.

## PEDIATRIC TRIALS: RESULTS

Concerning pediatric trials, the first example and one of the most cited, is a study of 1980 written by Oehling et al^30 ^that showed a reduction of symptoms severity using bacterial lysates but there is no control group: this study cannot be considered a correct example of RCT. Different clinical trials show negative results: for example in 1986 a study involving 825 children in the treatment group and 327 in the placebo one demonstrated that the administration of a bacterial lysate applied intranasally for 6 months did not reduce the number of acute respiratory diseases compared with placebo.^31^

Other studies, instead, revealed the benefits of bacterial lysates use. In a RCT where children with rhinosinusitis underwent a treatment with bacterial lysate (OM-85BV), a decrease in infectious episodes incidence and duration , their number and the duration of concomitant treatments; furthermore, the clinical response showed a correlation with an increase in serum levels of IgA (active 6.53 ŷ 0.96 vs placebo 6.81 ŷ 0.8).^32^

A trial involving 423 children attending day-care centers in which there were subjects with a higher risk of infection, showed that during the period of treatment with bacterial lysate, treated children had a relative lower risk to present 3 or more episodes of upper respiratory infections.^33^

In another study, 56 young patients affected by subacute sinusitis were involved in a RCT with the duration of 6 months, to evaluate the possible positive effects of bacterial lysates (OM- 85BV) added to amoxicillin/clavulanate. The results showed that the symptoms improved in the group that underwent the combination therapy, and these patients had a lower incidence of respiratory infections (active 1.56 ŷ 0. 3 vs placebo 2.22 ŷ 0. 43).^34^

Another RCT involving 188 pediatric patients put in evidence that the rate of infection was reduced by 50% in treated patients, and this protection was sustained for half a year after the end of drug administration; drug reactions were few, transient, expected and non serious.^35^

In another trial, 54 children aged 1-12 years were followed and divided in 2 groups (active/placebo). During the study number and duration of acute respiratory tract infections, and the number of antibiotic courses needed, were registered. The results showed a reduction in the number of infectious episodes, and a more significant reduction in both antibiotic requirements and duration of the infection episodes in the treated group compared with placebo (5.04 ŷ 1.99 vs 8.0 ŷ 2.55).^36 ^A RCT including 232 children aged 3-5 years showed that the treatment with PMBL significantly reduced the rate of upper respiratory tract infections (16% decrease in pharyngitis and otitis media) , being this reduction higher in children affected more frequently by this kind of infections; the drug was also safe and well tolerated compared with placebo (*P *` 0.05).^37^

A pilot study involving 89 children pointed out that the administration of PMBL could lead to a significant decrease in recurrent respiratory infections, compared with the control group represented by the same children during the previous year (7.84 vs 4.78, *P *` 0.05 with PMBL; 6.78 vs 4.78, *P *` 0.05 without PMBL); also phlogosis indexes were significantly lower in the treated group, and the values of B-lymphocytes were found to be increased (9.9 with PMBL vs 5.9 without PMBL).^38^

In another study involving patients from 3 to 6 years of age affected by recurrent respiratory infections and IgG deficiency a RCT proved the beneficial effect of bacterial lysate.^39 ^Others studies with young patients are in progress in these years; after this pediatric examples we try to analyze the results of bacterial lysate in trials with adult patients.

## ADULT TRIALS: RESULTS

The first studies of bacterial lysates in the field of respiratory diseases go back to the 1980s, when the bacterial extracts began to be considered and studied with more attention in the clinical practice. In 1989, Heintz et al designed a double-blind placebo controlled trial to test the efficacy of bacterial lysates in treating patients suffering from chronic purulent sinusitis. This study, carried over on 284 patients for a duration of 6 months, showed the efficacy of treatment in reducing purulent nasal discharge, cough, and headache, on the basis of the score of symptoms.^40^

A RCT performed in 1994 focused its attention on acute bronchitis and pneumonia, in 354 patients living in institutions for elderly; the aim of the trial was to assess the effects of using bacterial lysates in relation to lower respiratory tract infections incidence. The authors observed a reduction of 28% of infections, and this was because of a 40% reduction in episodes of acute bronchitis, with no differences in the incidence of pneumonia in the 2 groups. During the 6 months of duration of the trial, a larger number of patients in the active treatment group presented no episodes of acute bronchitis and a reduction of antibiotics needed in the same group observed.^41^

Another RCT involving 104 patients affected by chronic bronchitis, comparing bacterial lysates (OM-85BV) to placebo over a period of 6 month showed a significant reduction of the duration and symptoms of acute episodes in the treatment group, with a concomitant sparing-effect on the use of antibiotics, an increase in serum IgA levels and in T-lymphocyte counts.^42^

In a recent Italian study, 140 patients with a medical history of recurrent URTIs were randomly assigned to 3 groups: the first group received, by sublingual administration, a PMBL (Ismigen) obtained by mechanical lysis of 48 billion bacteria commonly responsible for upper respiratory tract infections; the second one received an oral immunoregulating lysate obtained by chemical lysis of 36 billion bacteria (CLBL); the third one, as control group, did not receive any immunostimulating treatment. The number of URTIs, was significantly lower in the PMBL group with respect to the other groups (*P *` 0.05). Furthermore, patients treated with PMBL group remained free from respiratory infections episodes, compared with the other groups. The duration of these episodes and the number of working days lost were significantly lower in the PMBL group during treatment and follow- up periods. None of the patients treated with PMBL needed concomitant assumption of antibiotics, while 9 patients of the CLBL group received such treatment (*P *` 0.05). Furthermore, no adverse events were described. The study showed the efficacy of the 2 treatments, but the best results were achieved with the use of PMBL in comparison to those achieved with placebo and CLBL.^43^

Similarly, another Italian study carried out in 47 nuns (aged 25-80 years) suffering from recurrent infections of the upper respiratory tract has found that during the treatment and the follow-up phases the number of respiratory infections and their duration were statistically significantly lower in the group treated with a PMBL, than in the placebo group. Furthermore, in the PMBL group a significant increase of serum immunoglobulins (IgG +35%; IgM +86%; IgA +80%) and salivary IgA (+110%) was observed, in comparison to baseline, while no significant differences were found in the placebo group. All these beneficial effects of bacterial lysates during the treatment period lengthened also during a 3 months follow-up and no adverse events related to the medication trial were described in both groups.^44 ^The clinical efficacy of bacterial lysates is confirmed also by another survey involving patients suffering from both respiratory infections and otitis media. As a matter of fact, the bacterial lysate treatment was associated with a significant reduction in the frequency and duration of infectious episodes, incidence of fever, ancillary therapies and number of working days-lost; furthermore, changes in both immunologic (such as increased serum concentrations of immunoglobulins) and auditory function parameters were observed.^45^

The efficacy of microbial extracts has been studied and demonstrated in chronic pulmonary illnesses, such as COPD. The most common and serious complications of COPD are represented by periodic exacerbations in which the role of bacterial infections is really important: *H. influenzae *is the causal agent in more than half of all bacterial exacerbations, *S. pneumoniae *and *M. catarrhalis *for a further third, but also Mycoplasmas and viruses are also involved. The rightful place of antibiotic therapy is debated and there are also many controversies and different opinions about the use of antimicrobial drugs, their real clinical efficacy and the possible adverse events. As a matter of fact, only 18-25% of patients receiving home therapy do not respond to initial treatment: these findings suggest that the respiratory pharmacokinetics could be suboptimal, or bacteria are becoming progressively drug-resistant or, finally, the antibiotic therapy is not completely effective or powerful.^46-48^

Stimulating the immune responses against pathogens could represent a powerful strategy to improve the quality of life of COPD patients. One of the first RCT on the use of bacterial lysates in COPD patients, lasting 6 months and involving 265 patients, demonstrated a statistically significant reduction of infectious events, and a concomitant reduction in the use of antibiotics.^49 ^Another RCT recruited 381 patients with COPD followed for a 6 months period. The risk of having one or more exacerbations during the 6 months period was similar in both groups, but the most significant results showed a clear reduction in the total number of days of hospitalization for respiratory problems in the group treated with bacterial lysate compared with the placebo group; in addiction, the overall risk of being hospitalized was reduced for the same patients group (16.2% vs 23.2%). Furthermore, the number of deaths observed was reduced in the treatment group (2 vs 6), but without statistical significance.^50 ^These results showed that immunostimulating agents could be useful for treating patients with COPD, being able to reduce the likelihood of severe respiratory events possibly responsible for hospitalization.

Another study involving 90 patients considered different endpoints: the frequency of acute exacerbations, lung function, parameters symptom scores, all analyzed during 1 year after the end of the treatment. The results showed that, in the group treated with bacterial lysate (OM-85BV) compared with the placebo group a decrease in incidence, duration, and severity of acute exacerbation occurred, and an improvement in symptom scored, a reduction in the course of antibiotics and a higher bacterial clearance rate in sputum cultures.^51^

A systematic review and metanalysis was published in 2004^52^; the central point of the study was the use of oral purified bacterial extracts in the treatment of patients affected by COPD. An extensive and systematic search for randomized clinical trials in all the electronic databases, biographies, and data from manufacturers was performed; a total of 13 studies, corresponding to almost 2000 patients, were included in the analysis. Concerning the prevention of exacerbations, the data were globally heterogeneous and the difference between active bacterial lysates and placebo did not reach statistical significance, but the metanalysis also showed a shorter duration of exacerbations in the active treated group and the improvement of respiratory symptoms, as assessed both by patients and physicians. Although this large systematic review did not find bacteria lysates to be beneficial in the prevention of COPD exacerbations, it did find trends toward shorter duration of exacerbation and reduction in hospitalization, with consequent important economic consequences. It is also important the benefit on symptoms as a reflection of a better quality of patients life.

In a double-blind multicenterd study, 178 patients were randomized into 2 different groups, one treated with PMBL and the other with placebo during a 3 months period, 10 day per month. At the end of the treatment, patients were clinically followed for 9 months. Selected clinical endpoints were seen to be significantly lower in the group treated with the lysate than in the placebo group: the exacerbations frequency (215 vs 248 cases) and duration (10.6 vs 15.8 days) , antibiotic consumption (270 doses) and hospitalization time (275 vs 590 days).^53 ^In another study, it was investigated the PMBL addition to therapy for COPD patients consisting in salmeterol/fluticasone, 63 patients were randomly divided in 2 groups (A and B); all patients were treated with salmeterol/fluticasone 50/500 mg bid.

Thirty-three subjects also received one capsule of PBML daily the first 10 days of 3 consecutive months (group B). This treatment was repeated 3 months after the end of the first course. During the study, respiratory symptoms (such as amount and color of sputum, severity of dyspnoea, frequency of cough, fever), concomitant medications (antibiotics and oral corticosteroids), and hospitalization were registered. At the end of 12 months of follow-up it was clear that PBML had reduced the total number of exacerbations (23 of 30 patients in group A and 21 of 33 patients in group B), the number of exacerbations per patient per year (0.67 in group A and 0.54 in group B); the number of exacerbations that needed treatment with oral corticosteroids (52% in group A and 43% in group B) and the rate of hospitalization (4/30 [0.13] in group A and 3/33 [0.09] in group B). There were no significant differences between the 2 groups regarding the exacerbation symptoms, but a decreased need of antibiotics was observed in group B.^54^

## DISCUSSION AND CONCLUSION

Both in pediatric and adult trials, a positive trend in the results can be found: a reduction in infection rates and duration and use of antibiotics, plus a beneficial effect on symptoms. We have to bear in mind there are also new clinical perspectives and approaches about the potential use of bacterial lysates in different pathologies, besides the common respiratory illnesses, with possibility to improve the patient's quality of life in several different diseases. In conclusion, although these findings are encouraging, more RCT are needed to further elucidate the mechanism of action of bacterial lysates.

According with Braido et al^18 ^we need further trials including a higher number of selected patients and well-designed trials in term of blinding and randomization procedures. These studies will help us to find stronger evidence of bacterial lysates beneficial effects on symptoms improvement, infections prevention and, most of all, on quality life of our patients. A further analysis of mechanism of actions of bacterial lysates on animals and humans are ongoing and needed.
